# Implementation and Outcomes of a Pilot Collaborative Surgical Hydrocele Training in Côte d’Ivoire

**DOI:** 10.4269/ajtmh.23-0554

**Published:** 2023-11-13

**Authors:** Kevin Montes, Georgina Angoa, Catherine M. Bjerum, Adama Guira, Olivier K. Kouadio, Allassane F. Ouattara, Gary J. Weil, Peter U. Fischer, Aboulaye Meite, Benjamin G. Koudou, Philip J. Budge

**Affiliations:** ^1^ Infectious Diseases Division, Department of Medicine, Washington University School of Medicine, St. Louis, Missouri;; ^2^ Centre Suisse de Recherches Scientifiques en Côte d’Ivoire, Abidjan, Côte d’Ivoire;; ^3^ Center for Global Health and Diseases, Case Western Reserve University, Cleveland, Ohio;; ^4^ Department of Surgery, Hopital Saint Camille, Nanoro, Burkina Faso;; ^5^ Université Nangui Abrogoua, Abidjan, Côte d’Ivoire;; ^6^ National Neglected Tropical Diseases Control Program, Ministry of Public Health and Hygiene, Abidjan, Côte d’Ivoire

## Abstract

Lymphatic filariasis (LF) is a neglected tropical disease that can cause hydrocele and its associated stigma, loss of economic productivity, and depression. Hydrocele surgery is an essential part of LF morbidity management but can be difficult for national programs to implement. To improve access to hydrocele surgeries in Côte d’Ivoire, we provided a WHO-certified surgical training for six surgical teams from five health districts in Côte d’Ivoire. We then evaluated the surgical outcomes and assessed the impact of hydrocele surgery on quality of life of hydrocelectomy patients. Preoperative and operative records were reviewed to describe baseline hydrocele characteristics and operative details. Postoperative interviews were conducted 4 to 6 months after surgical correction using a standardized questionnaire. Seventeen men underwent surgery during the training and were available for an interview at the 6-month visit. At the time of 6-month follow-up, 11/17 (64.7%) reported improvement in activities of daily living and reduction in difficulties with work, 8/17 (47.1%) reported an improved economic situation, 15/17 (88.2%) reported improved social interactions, and 15/16 (93.8%) reported improved sex life after surgical correction. Three patients (17.6%) had minor postoperative complications, but none required hospitalization. All 17 patients who were available for an interview were satisfied with their surgery. Surgical hydrocelectomy training in Côte d’Ivoire was well received and provided life-altering health improvements for participating patients across multiple domains of life. Support to scale up surgical capacity for this neglected problem is needed.

## INTRODUCTION

Lymphatic filariasis (LF) is a mosquito-borne neglected tropical disease (NTD) endemic in 72 countries in Asia, Africa, Oceania, and the Americas.^1^ It is caused by the threadlike (filarial) parasites *Wuchereria bancrofti*, *Brugia malayi*, and *Brugia timori.* After infection, these parasites mature and form worm nests within host lymphatic vessels, leading to lymphatic dysfunction and lymphedema. The Global Program to Eliminate Lymphatic Filariasis (GPELF) proposed two strategies to eliminate LF as a public health problem: interrupting transmission through mass drug administration (MDA) and morbidity management and disability prevention. Since the advent of GPELF in 2000, there has been a significant reduction in the number of infected individuals, with 51.4 million individuals worldwide remaining infected as of 2021.^1^ Approximately one in three infected individuals eventually develops clinical symptoms. In men, the most common symptom of bancroftian filariasis is hydrocele, which develops from accumulation of fluid in the tunica vaginalis of the scrotum.^2^ Men affected by hydrocele experience disfigurement, loss of self-esteem, impaired sexual function, and loss of social standing.^3^^–^^5^ In addition to these physical and social consequences, LF causes a significant economic burden that perpetuates poverty by reducing productivity and increasing healthcare spending by those afflicted.^6^^,^^7^

Community-wide MDA has had a major impact in reducing the transmission of LF,^1^^,^^8^ preventing onset of disability or disfigurement in millions. However, MDA does not reverse chronic morbidity in affected persons. Surgery is the definitive management for hydrocele, and provision of hydrocelectomy is recommended under WHO LF morbidity management guidelines.^9^ However, despite being recognized by the WHO as an essential surgery, hydrocelectomy is not yet available at the scale required to alleviate the suffering from this important public health problem. Relatively little has been published regarding patient satisfaction after surgical correction of hydroceles.^10^^,^^11^

Over the past several years, we have conducted several collaborative LF clinical trials and field studies in Côte d’Ivoire.^12^^–^^14^ To facilitate access to hydrocele surgery for participants in these studies and for others in the community needing hydrocelectomy, we partnered with the National Program for Schistosomiasis, Soil Transmitted Helminthiasis and Lymphatic Filariasis Control (PNL-SGF) in Côte d’Ivoire to support a 5-day hydrocelectomy training for surgical teams from Agboville and surrounding districts. This provided an opportunity to document the clinical and quality of life outcomes of hydrocelectomy surgery in patients who received hydrocelectomies as part of this training.

## MATERIALS AND METHODS

**Figure 1.**
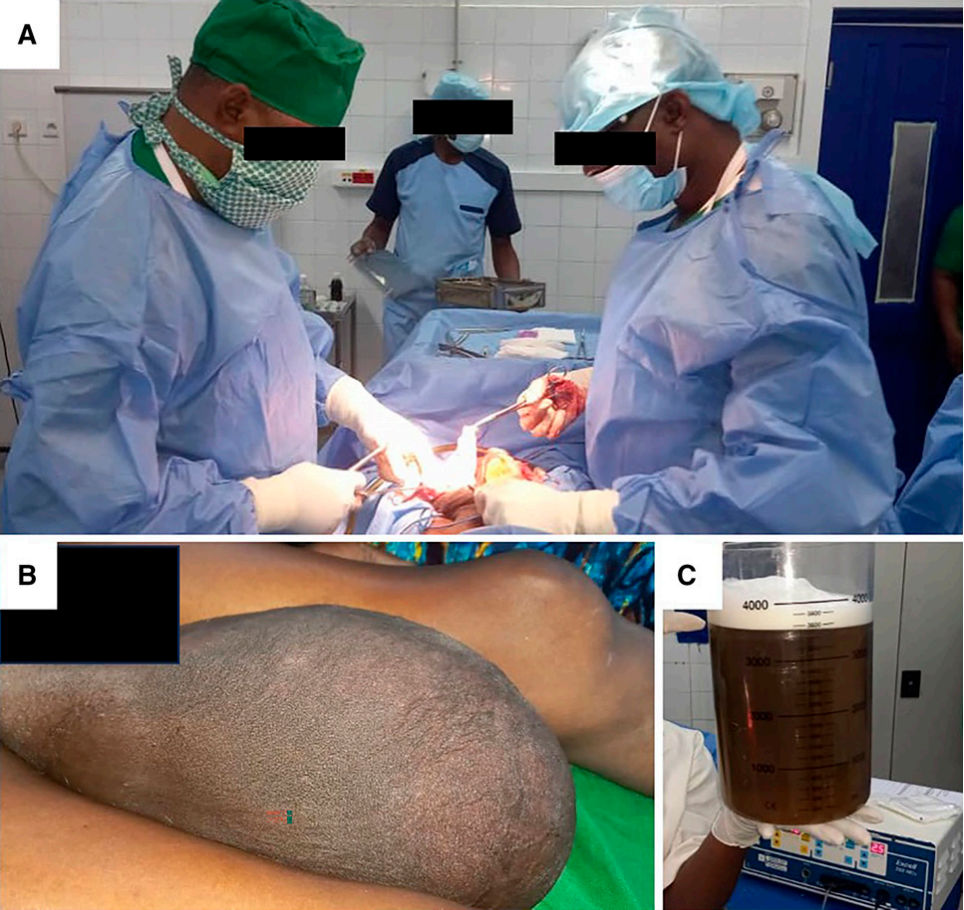
Intraoperative training on surgical hydrocele repair. (**A**) Surgical team at work. (**B**) One example of a large hydrocele that was repaired. (**C**) Suction canister showing the large volume of fluid removed from a single large hydrocele.

From February 21 to 25, 2022, PNL-SGF coordinated surgical training for physicians, nurses, and operating staff from the Akoupé, Agboville, Bongouanou, Gagnoa, and Tiassalé health districts. A WHO-certified instructor from Burkina Faso, A. G., conducted the training, which included 1 day of classroom learning, 2 days of instruction in the operating theater, and 2 days of instruction on postoperative care, according to WHO protocols (Figure 1).^15^ Fifteen surgeons received training during this time frame. The surgical training cost was 4,828,592 francs CFA (approximately $8,000 USD), and the total cost of all 17 surgeries was 2,966,592 francs CFA (approximately $5,000 USD). A morbidity survey to identify men needing hydrocelectomy had been conducted by the NTD program in the neighboring Bongouanou District in 2020–2021. Men identified in this survey as having clinical hydroceles needing surgery and considered eligible by the attending surgeon were offered the opportunity to receive surgery during the training. The present study was conducted 4 to 6 months after surgery to assess their experience and change in quality of life.

### Study procedures.

To collect data on postsurgical outcomes, we used a modified survey developed by the Burkina Faso LF program with the technical and financial support of Helen Keller International (Supplemental File 1). The survey collected basic demographic information, clinical information regarding the hydrocele, surgical technique, and a quality of life questionnaire assessing employment and social and economic wellness. A member of the study team interviewed participants between 4 and 6 months after surgery and reviewed operative records. Deidentified data were entered into a secure REDCap electronic database hosted at Washington University in St. Louis.^16^^,^^17^

### Definitions.

Hydrocele staging and grading were conducted according to the clinical classification proposed by Capuano and Capuano.^18^ *Early postoperative complication* was defined as a complication within 5 days of surgery.

## RESULTS

**Figure 2.**
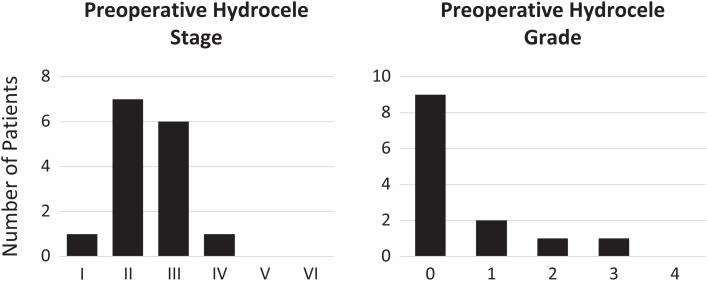
Hydrocele stage and grade before surgery among the 15 and 13 patients, respectively, for whom information was recorded.

Twenty-six men were evaluated for surgery as part of the training. Nine were excluded, four because of comorbid conditions, four because of misdiagnosis of hydrocele, and one who refused consent for surgery. All 17 individuals who underwent surgical repair of their hydrocele were available for an interview and were included in this analysis (Table 1). Median participant age was 50 years (range, 38–76 years). Ten of the men were married, three were widowers, and four were single. Eleven of the men had no formal education, and 15 worked in agriculture. Preoperative hydrocele stage and grade were recorded for 15 of 17 patients (Figure 2). All but one of the hydroceles were grade 2 or larger (larger than a tennis ball), and seven extended below the mid-thigh (grade 3 or higher). Seven of the men had bilateral hydroceles, and five had a hydrocele and concomitant hernia.

**Table 1:** Demographic information of surgery participants

Demographic information	Survey response
Age in years, median (range)	50 (38–76)
Married, *n* (%)	10 (58.8)
No formal education, *n* (%)	11 (64.7)
Agricultural worker, *n* (%)	15 (88.2)
Hydrocele associated with a hernia, *n* (%)	5 (31.3)
Bilateral hydroceles, *n* (%)	7 (43.8)

### Surgical technique, postoperative complications, and hydrocele recurrence.

Information regarding surgical technique used was available for 13 patients, all of whom underwent excision/resection of the hydrocele (Table 2). Herniorrhaphy was performed simultaneously in four patients. All participants were followed up as inpatients for at least 2 days postoperatively. No early postoperative complications were identified. Two patients returned to the hospital postoperatively; one patient had poor wound healing and the other poorly controlled postoperative pain. However, neither required readmission. No other complications were identified. There were no recurrences of hydrocele noted at the time of the postoperative interviews.

**Table 2:** Surgical outcomes and postoperative assessment

Chart review	Result
Surgical information		
Duration of surgery in minutes, mean	74.4
Duration of hospitalization after surgery in days, median (IQR)	2 (2–7)
Required drain placement, *n* (%)	2 (11.7)
Early surgical complication,* *n* (%)	1 (5.8)
Any surgical complication, *n* (%)	3 (17.6)
Postoperative assessment, *n* (%)	
Improved economic situation after surgery	8 (47.1)
Improved social interactions after surgery	15 (88.2)
Improved sex life after surgery	15 (93.8)
Satisfied with surgery	17 (100)
Would recommend surgery to others	16 (94.1)

IQR = interquartile range.

* *Early surgical complication* was defined as a complication within the first 5 days of surgery.

### Activities of daily living and employment.

**Figure 3.**
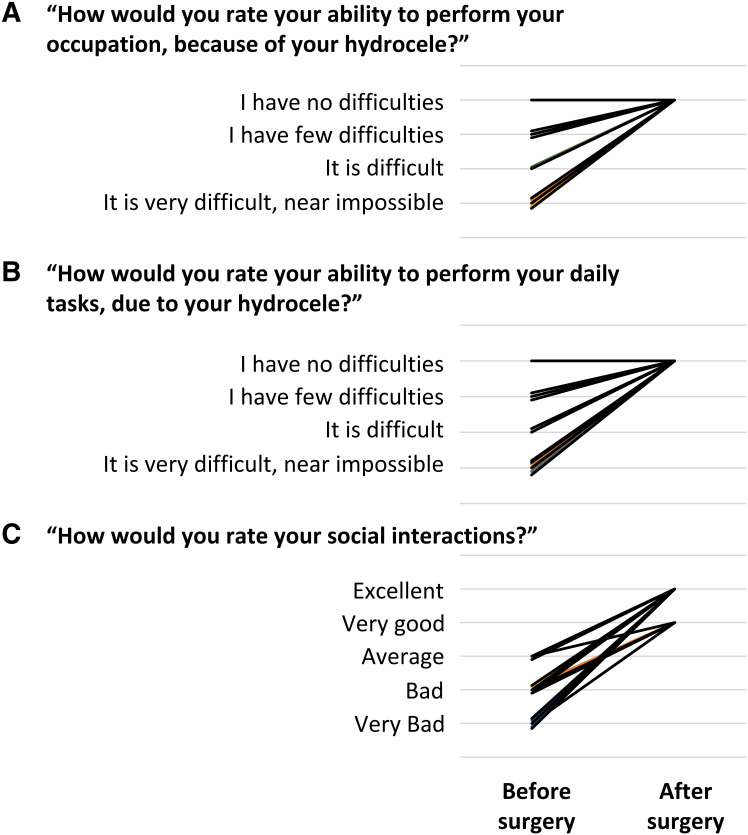
Changes in self-assessment in difficulty with work, activities of daily living, and social interactions before and after surgery. (**A**) Change in difficulty with work. (**B**) Change in difficulty with activities of daily living. (**C**) Change in social interactions. Each line corresponds to an individual. For **A** and **B**, individuals who answered “I don’t know” were excluded from the graph.

Fifteen of the 17 men (88%) described at least some amount of preoperative difficulty with activities of daily living or with employment that they attributed to their hydrocele (Figure 3). Of these men, 11 endorsed missing days of work because of their hydrocele (an average of 2.5 days of work per week). After surgery, 11 of the 15 men (73%) who initially expressed hydrocele-related difficulties with activities of daily living or employment reported resolution after surgery.

### Social interactions, sexual health, and overall satisfaction with surgery.

Before surgery, 11 of the 17 participants (65%) reported bad or very bad social interactions and described being subject to ridicule and teasing from friends or potential sexual partners. After surgery, all 11 men reported improvement in their social interactions (Figure 3). Fifteen of 17 participants endorsed improvement in their sex life after undergoing surgery and described an improvement in their erections, more pleasurable intercourse, and loss of shame in having sex. All 17 reported that they were very satisfied with their surgery. Only one of the 17 participants responded that he would not recommend hydrocele surgery; he commented that this was because of fear in his community that he would die because of the surgery.

## DISCUSSION

Although MDA programs have brought significant improvements in the prevalence of LF, there is a significant unmet need for morbidity management for men with filarial hydroceles. Challenges to scaling up hydrocele surgeries include cost, transportation of patients to medical centers capable of performing surgery, lack of trained surgeons in areas with high LF burden, and logistical difficulties with patient follow-up.^19^

Prior to the start of MDA, the global economic burden of LF was estimated at $5.77 billion (USD) annually, or $114.69 per case of chronic LF.^7^ In northern Ghana, for example, approximately 7% of the potential male labor force was estimated to be lost because of chronic filariasis.^20^ Hydroceles may reduce an individual’s annual productivity by 15–19%. In addition, each episode of acute dermatolymphangioadenitis, an acute inflammatory exacerbation of chronic LF, reduces productivity between 77% and 100% for a period of weeks.^6^^,^^7^ In our study, nearly all men surveyed endorsed some amount of difficulty with employment before surgery; three-quarters of the respondents missed an average of 2.6 days of work per week, and most described resolution of this difficulty after hydrocelectomy. Scaling-up of surgical capacity has the potential of bringing significant economic improvement to affected populations.

Sexual dysfunction is a common complaint of men affected by hydroceles. Marriages without sexual intimacy or with dyspareunia, shame about disfigurement, and suicidal ideation have been described.^5^^,^^10^ In our study, all but one of the individuals endorsed an improvement in sexual function after hydrocelectomy. The individual who reported no improvement in sexual function was a widower, and the surveyor did not inquire whether the individual was or intended to be sexually active.

Overall, we found high levels of patient satisfaction after hydrocele surgery. A prior study of patients in Ghana who underwent hydrocele surgery similarly noted improvements in health, work capacity, and sexual performance.^10^ In a more recent study, Malawian men with stage II or III and grade 0 or 1 hydrocele who underwent hydrocelectomy by local surgeons reported that their presurgical impairments (pain, mobility, difficulties in usual activities, self-care, social issues, or psychological health) had total resolution by 6 months after surgery. Before surgery, a majority of patients had missed days of work (with approximately one in four missing more than 10 days per month), with resolution of these difficulties postoperatively.^11^ These studies highlight the urgent need for expanding capacity to address this important public health problem.

Current barriers to widespread availability of hydrocelectomy include lack of surgical capacity, cost, transportation and geographic isolation, treatment avoidance, and lack of awareness of the possibility of surgery.^11^^,^^21^^,^^22^ Building local capacity, as was done in this region of Côte d’Ivoire, will be key to addressing long-term morbidity in the areas most affected by LF. Community concerns of increased morbidity or mortality after hydrocele surgery were the rationale given by the only patient who would not recommend surgery to others; similar concerns were noted among individuals in Ghana.^10^ This suggests that improvements in surgical capacity should be accompanied by public health messaging about the benefits and safety of hydrocele surgery in affected communities. In the months after this training, four additional patients had hydrocele surgery performed by surgeons who participated in this training, and an additional 12 procedures are currently planned.

Our study has several important limitations. The study was small and retrospective, and the surgical details and postoperative data for some patients are incomplete. Because the survey was not implemented preoperatively, each individual’s self-assessment of difficulties with activities of daily living and work, quality of social interactions, etc., was subject to recall bias. Despite these limitations, our study suggests that building surgical capacity can lead to well-tolerated surgery and improvements in quality of life across various important domains.

## Supplemental files

10.4269/ajtmh.23-0554 Supplemental Materials
