# Supplementary material for: Implementation and Outcomes of a Pilot Collaborative Surgical Hydrocele Training in Côte d’Ivoire
Source: Am J Trop Med Hyg. 2023 Nov 13;110(1):194–8. doi: 10.4269/ajtmh.23-0554 (PMC10793025; doi:10.4269/ajtmh.23-0554)
Supplement: Supplemental Materials [file tpmd230554.SD1.pdf]

QUESTIONNAIRE POUR LE SUIVI POST-OPÉRATOIRE DES PATIENTS OPERES D'HYDROCELE

I. Informations générales

DRS : ……………………… District : ………………… Formation sanitaire : ………………….
Village/Secteur : ………………………….. Date de l'enquête : ______/_____/______
Evaluateurs :……………………………………………………………………………………
……………………………………………………………………………………
……………………………………………………………………………………

II. Informations sur l'hydrocèle opérée et examen clinique

Indentification du patient *(questions à poser au patient)*

- Prénom et nom du patient : ……………………………………….
- Age (années) : ………………….
- Situation matrimoniale du patient
  ☐ Marié ☐ Divorcé ☐ Célibataire ☐ Veuf
- Niveau d'instruction ?
  ☐ Primaire ☐ Secondaire ☐ Supérieur ☐ Alphabétisé ☐ Aucun

Quel était le diagnostic du patient ? *(à compléter grâce au dossier du patient)*

☐ Hydrocèle gauche ☐ Hydrocèle droite ☐ Hydrocèle bilatérale

☐ Hydrocèle associé à une hernie ☐ Information non disponible

Quelle est la technique qui a été pratiquée ? *(à compléter grâce au dossier du patient)*

☐ Excision/Résection ☐ Éversion ☐ Herniorraphie ☐ Orchidectomie

☐ Autre *(Veuillez expliquer)* …………………………………… ☐ Information non disponible

Informations sur la chirurgie

- Nom du chirurgien : ……………………………………….
- Durée de la chirurgie : …………. Minutes ☐ Information non disponible
- Pose de drain : ☐ Oui ☐ Non
- Taille / stade de l'hydrocèle :…………………. ☐ Information non disponible
- Grade de l'hydrocèle : ………… ☐ Information non disponible
- Durée du séjour à l'hôpital : ……………… jours
- Est-ce que le registre de chirurgie indique des examens du suivi postopératoire ?
  ☐ Oui ☐ Non ☐ Information non disponible
  Si oui, à quel(s) moment(s) après l'opération ? (*précisez le nombre et encerclez jours ou mois* :
  ………………..Jours/Mois ………………Jours/Mois
  ………………..Jours/Mois ………………Jours/Mois
  ………………..Jours/Mois ………………Jours/Mois

- Entre les jours 0 à 5, le patient a-t-il eu des complications ? (*à compléter grâce au dossier du patient*)

  ☐ Information non disponible ☐ Pas de complications ☐ Hémorragie
  ☐ Hématome ☐ Infection ☐ Autre complication *(A préciser)* ……………………………………......

- Si le patient a eu une complication, quel a été la conduite à tenir ? *(à compléter grâce au dossier du patient)*

☐ Antibiotiques ☐ Chirurgie ☐ Patient garde en hospitalisation
☐ Mise en observation ☐ Information non disponible
☐ Autre *(Veuillez expliquer)* ……………………………………………………………………………………

- Si le patient a eu une complication, quel a été le résultat final ? *(à compléter grâce au dossier du patient)*

☐ Information non disponible ☐ En cours de traitement ☐ Résolue ☐ Dommages permanents
☐ Aggravation de l'état général du patient (*préciser*) : ……………………………………………………………
☐ Décès (*préciser le diagnostic*) : …………………………………………………………………………...
☐ Autre *(Veuillez expliquer)* …………………………………………………………………………………..

III. Examen clinique du patient

**Observations**	**DROITE**		**GAUCHE**	
	Oui	Non	Oui	Non
Taille des bourses après intervention à 1 mois	Circonférence :………...cm *(le plus grand périmètre)*<br>Longueur :……/…………cm *(base de la verge au point le plus bas)*			
Taille des bourses après intervention à 4 mois	Circonférence :………...cm *(le plus grand périmètre)*<br>Longueur :……/…………cm *(base de la verge au point le plus bas)*			
Récidive *(collection liquide dans scrotum-> présence d'hydrocèle. Si bilatérale, cochez les deux colonnes)*				
Autre anomalie *(à préciser)*				
Autre complication : ____ Oui ; _____Non<br>Si oui, expliquez :				
**Observations générales**				
**Conclusions**				

IV. Informations sur l'intervention *(questions à poser au patient)*

- Comment avez-vous eu l'information sur la chirurgie : ……………………………………………
- Qui vous a conseillé de vous faire opérer ? ……………………………………………………
- Combien de temps êtes-vous resté à l'hôpital après votre opération ? ……………jours
- Suite à l'opération, êtes-vous retourné à l'hôpital pour un problème au niveau de votre hydrocèle ?

  ☐ 1. Oui ☐ 2. Non

  Si oui, pourquoi ? (NE LISEZ PAS AU PATIENT) :

  Hémorragie ☐ Hématome ☐ Infection ☐

  Autre complication *(Veuillez expliquer)* ……………………………………………………………

- Avez-vous été réadmis en hospitalisation for complications of the hydrocele surgery ?

  ☐ 1. Oui ☐ 2. Non

V. Satisfaction du patient *(questions à poser au patient)*

- Quel est votre occupation actuelle? ………………………………………………………………..
- Y-a-t-il des jours où vous ne pouvez pas travailler **à cause de votre opération** actuellement?

  ☐ Oui ☐ Non ☐ Ne sait pas

  *Si oui, combien de jours au cours de la dernière semaine n'avez-vous pas pu travailler **à cause de votre opération?*** …………………………. jours

- Est-ce que votre occupation actuelle est la même que celle avant votre chirurgie ?

  ☐ Oui ☐ Non

  Si non, quel était votre occupation avant la chirurgie de l'hydrocèle ? …………………………………..

- Aviez-vous déjà eu des jours où vous ne pouviez pas travailler à cause de votre hydrocèle, avant votre opération?

  ☐ Oui ☐ Non

  Si oui, en moyenne, combien de jours par semaine pourriez-vous ne pas travailler en raison de votre hydrocèle? ……………………………jours

- Avant l'intervention, comment évaluez-vous votre capacité à accomplir votre occupation, en raison de votre hydrocèle ? :

  ☐ C'était très difficile, voire impossible ☐ C'était difficile ☐ J'avais peu de difficultés

  ☐ Je n'avais aucune difficulté ☐ Ne sait pas

  Si la réponse indique les difficultés : Quelles taches étaient difficiles ?

  ………..

- Comment évaluez-vous votre capacité à accomplir votre occupation depuis que vous avez été opéré?

  ☐ C'est très difficile, voire impossible ☐ C'est difficile ☐ J'avais peu de difficultés

  ☐ Je n'ai aucune difficulté ☐ Ne sait pas

  *Si la réponse indique les difficultés, d*emandez de préciser la (les) difficulté (s)?

  ………..

- Pensez-vous que la chirurgie vous a aidé à changer votre situation économique ?

☐ Oui ☐ Non ☐ Ne sait pas

- Si oui, comment voyez-vous le changement dans votre situation économique que la chirurgie a créé ?

☐ Amélioration ☐ Détérioration ☐ Ne sait pas

Expliquez : ……………………………………………………………………………………………………….

- Avant l'intervention, comment évaluez-vous votre capacité à accomplir vos tâches quotidiennes en raison de votre hydrocèle ? :

☐ 1. C'était très difficile, voire impossible ☐ 2. C'était difficile ☐ 3. J'avais peu de difficultés

☐ 4. Je n'avais aucune difficulté ☐ 5. Ne sait pas

- Comment évaluez-vous votre capacité à accomplir vos tâches quotidiennes depuis que vous avez été opéré ? :

☐ 1. C'est très difficile, voire impossible ☐ 2. C'est difficile ☐ 3. J'avais peu de difficultés

☐ 4. Je n'ai aucune difficulté ☐ 5. Ne sait pas

- Est-ce que vous pensez que la chirurgie vous a aidé à changer vos interactions sociales ?

☐ 1. Oui ☐ 2. Non ☐ 3. Ne sait pas

- Si oui, comment voyez-vous le changement dans vos interactions sociales que la chirurgie a créé ? :

☐ 1. Amélioration ☐ 2. Détérioration ☐ 3. Ne sait pas

Expliquez : ……………………………………………………………………………………………………..

- Avant l'intervention, comment évalueriez-vous vos interactions sociales ?

☐ 1. Excellent ☐ 2. Très bon ☐ 3. Moyen

☐ 4. Mauvais ☐ 5. Très mauvais

- Comment évalueriez-vous vos interactions sociales depuis que vous avez été opéré ?

☐ 1. Excellent ☐ 2. Très bon ☐ 3. Moyen

☐ 4. Mauvais ☐ 5. Très mauvais

- Est-ce que votre vie sexuelle a changé depuis la chirurgie ?

☐ 1. Oui, amélioration ☐ 2. Oui, détérioration ☐ 3. Non, pas de changement

Précisez le changement : ……………………………………………………………………………………..

- Quelles sont vos pensées générales au sujet de la chirurgie?

☐ 1. Je suis content que j'ai fait l'opération

(*Expliquer* : ……………………………………………………………………………………………..)

☐ 2. Je ne sais pas si je suis content d'avoir fait l'opération ou non

(*Expliquer* : ……………………………………………………………………………………………….)

☐ 3 J'aurais aimé ne pas avoir subi l'opération

(*Expliquer* : ………………………………………………………………………………………….)

- Etes-vous satisfait de votre opération ?

☐ Très satisfait ☐ Peu satisfait ☐ Insatisfait

- Recommanderiez-vous à quelqu'un atteint d'hydrocèle de se faire opérer ?

☐ 1. Oui ☐ 2. Non ☐ 3. Indifférent ☐ 4. Ne sait pas

Pourquoi ?...

<u>Commentaires</u> :……………………………………………………………………………………………………
………..
………

≈JE VOUS REMERCIE≈

Ministère de la Santé

MMDP PROVIDING A NEW CHANCE AT LIFE

TABLEAU RECAPITULATIF DES RESULTATS DE L'EVALUATION (par district)

**Dates**	
Dates exactes des périodes de chirurgie:	
Dates exactes du suivi post opératoires :	
**Echantillonnage**	**No. de personnes**
Nombre total personnes opérées pendant les périodes de chirurgie	
Nombre de personnes sélectionnées pour le suivi postopératoire *(selon le protocole, avant des replacements)*	
Nombre de personnes trouvées et examinées	
Nombre de personnes trouvées mais qui n'ont pas été examinées *(inclure ceux qui n'ont pas consenti à la participation au suivi)*	
Nombre de personnes absentes *(inclure ici toutes les personnes absentes, même s'ils ont été remplacés)*	
**Résultats de l'examen**	**No. de personnes**
Pas de complications	
Nombre de personnes présentant un problème de cicatrisation	
Nombre de personnes présentant une infection	
Nombre de personnes présentant une hydrocèle recrudescente	
